# Ambulance service recognition of health inequalities and activities for reduction: An evidence and gap map of the published literature

**DOI:** 10.29045/14784726.2024.6.9.1.47

**Published:** 2024-06-01

**Authors:** Fiona Bell, Ruth Crabtree, Caitlin Wilson, Elisha Miller, Rachel Byrne

**Affiliations:** Yorkshire Ambulance Service NHS Trust ORCID iD: https://orcid.org/0000-0003-4503-1903; Yorkshire Ambulance Service NHS Trust; Yorkshire Ambulance Service NHS Trust ORCID iD: https://orcid.org/0000-0002-9854-4289; NIHR Coordinating Centre ORCID iD: https://orcid.org/0000-0003-4729-8572; St Gemma’s Hospice

**Keywords:** healthcare disparities, health inequities, literature review, paramedics

## Abstract

**Background:**

Emergency medical services (EMS) are often patients’ first point of contact for urgent and emergency care needs. Patients are triaged over the phone and may receive an ambulance response, with potential conveyance to the hospital. A recent scoping review suggested disparities in EMS patient care in the United States. However, it is unknown how health inequalities impact EMS care in other developed countries and how inequalities are being addressed.

**Objectives:**

This rapid evidence map of published literature aims to map known health inequalities in EMS patients and describe interventions reducing health inequalities in EMS patient care.

**Methods:**

The search strategy consisted of EMS synonyms and health inequality synonyms. The MEDLINE/PubMed database was searched from 1 January 2010 to 26 July 2022. Studies were included if they described empirical research exploring health inequalities within ambulance service patient care. Studies were mapped on to the EMS care interventions framework and Core20PLUS5 framework. Studies evaluating interventions were synthesised using the United Kingdom Allied Health Professions Public Health Strategic Framework.

**Results:**

The search strategy yielded 771 articles, excluding duplicates, with two more studies added from hand searches. One hundred studies met the inclusion criteria after full-text review. Inequalities in EMS patient care were predominantly situated in assessment, treatment and conveyance, although triage and response performance were also represented. Studies mostly explored EMS health inequalities within ethnic minority populations, populations with protected characteristics and the core issue of social deprivation. Studies evaluating interventions reducing health inequalities (n = 5) were from outside the United Kingdom and focused on older patients, ethnic minorities and those with limited English proficiency. Interventions included community paramedics, awareness campaigns, dedicated language lines and changes to EMS protocols.

**Conclusions:**

Further UK-based research exploring health inequalities of EMS patients would support ambulance service policy and intervention development to reduce health inequality in urgent and emergency care delivery.

## Introduction

Emergency medical services (EMS) are often patients’ first point of contact for urgent and emergency care needs. Eleven NHS ambulance services provide EMS in England and can be accessed free at the point of care by calling the 999 emergency number. Callers to 999 are initially triaged by one of two dispatch systems, with lower acuity calls potentially receiving telephone advice only (‘hear-and-treat’) (Carter, 2018). In 2021/2022, over 10.5 million calls to the 999 emergency number were answered, with 7.8 million patients receiving a face-to-face response from an ambulance crew, only 58% of which resulted in conveyance to an emergency department (NHS Digital, 2023).

Alongside several organisational and system factors, O’Cathain et al. (2018) found that patients living in an area of social deprivation were more likely to receive ‘hear-and-treat’ telephone advice and, if attended by an ambulance crew, more likely to be conveyed to the hospital. Evidence from other areas of healthcare supports the notion that certain groups in society may experience differences in health and healthcare provision, known as health inequalities, which are unfair, avoidable and not wholly attributable to clinical criteria (e.g. presenting complaint, severity) or organisational pressures (e.g. available resources, ability to make own way to the hospital) (NHS England, 2023a).

The COVID-19 pandemic highlighted the impact of health inequalities on health outcomes (NHS Confederation, 2022; Suleman et al., 2021). To prioritise the most deprived and at-risk populations, NHS England (2021) developed the Core20PLUS5 framework, which seeks to inform action to reduce healthcare inequalities at both national and system level. The ‘Core20’ are the most deprived 20% of the national population and ‘PLUS’ population groups, which should be identified at a local level but may include ethnic minority communities, people with learning disabilities and those with shared protected characteristics. The framework focuses on five key clinical areas requiring accelerated improvement. The nuances of the five key clinical areas are not necessarily applicable to EMS patient care (e.g. maternity care continuity, annual mental health checks, cancer diagnosis and hypertension management).

A recent scoping review suggested that disparities exist in patient care from ambulance services in the United States (US) (Farcas et al., 2022). However, it is unknown how health inequalities impact the care provided by ambulance services in other countries, such as the UK, and how health inequalities are being addressed in line with the Core20PLUS5 framework. The objective of this study is to map out and highlight gaps in the published literature regarding health inequalities of EMS patients and interventions that have sought to reduce health inequalities within EMS.

## Methods

Due to the paucity of evidence on health inequalities within EMS and a desire to inform policy, an evidence gap and map study design was chosen. Evidence and gap maps involve systematically searching a field of published evidence to identify gaps in knowledge and future research needs (Miake-Lye et al., 2016) and to support commissioners or decision-makers by providing a visual overview of interventions and outcomes relevant to a particular topic (Saran & White, 2018; Snilstveit et al., 2016). The protocol for this study was not published. Relevant items of the Preferred Reporting Items for Systematic Reviews and Meta-Analyses (PRISMA) equity checklist were used to guide study reporting (Welch et al., 2012).

### Search strategy

Two authors, experienced in ambulance service research (FB) and public health (RC), developed the search strategy. The search addressed two facets (ambulance services and health inequalities). It involved searching titles and abstracts for the following keywords:

(paramedic* OR ambulance* OR EMS OR ‘Emergency Medical Service*’ OR ‘Emergency Medical Technician*’ OR ‘Emergency Nurse*’) AND (inequal* OR inequit* OR disparit* OR depriv*).

Searches were conducted in the MEDLINE/PubMed database and last updated on 26 July 2022. Studies that the authors were already aware of but were not retrieved through the database searches were also added manually.

### Study inclusion and exclusion criteria

English-language primary studies of qualitative, quantitative and mixed-methods methodology were included if they described health inequalities within ambulance service care (Table 1). Studies from low- or middle-income countries were excluded, as ambulance services in these countries were considered conceptually different from those in developed countries. We further excluded grey literature, evidence reviews and conceptual papers. The search was limited to studies published after 1 January 2010 for pragmatic reasons to reduce the number of retrieved articles and screening time, and out of a desire to synthesise contemporary evidence.

**Table 1.** Criteria for study inclusion.

Inclusion criteria	
Date	Evidence published from 1 January 2010 to 26 July 2022
Setting	Direct involvement by the ambulance service in the study
Population	No restrictions on population – included both adult and paediatric studies
Study type	No restrictions on study design
Model of care	Delivered directly by the ambulance sector
Other	Studies undertaken in developed countries, published in the English language
Exclusion criteria	
Study type	Papers that only describe systematic reviews, or reviews of other types; conceptual papers and projected possible future developments
Other	Studies conducted in low- or middle-income countries

### Study selection and data extraction

All titles and abstracts generated by the database search and added from other sources were downloaded and exported into Microsoft Excel (Version 365, Microsoft Corporation). Duplicate studies were removed manually. Study selection followed the conventional two-step process involving title and abstract screening and full-text review. The screening was undertaken independently on all studies by two reviewers (EM, RB) following the eligibility criteria. Any reviewer disagreements were resolved by consulting a third reviewer (FB). Two reviewers (FB, CW) jointly undertook data extraction of the included studies. The extracted items included descriptive information about the study (author, year, title, country, data collection period, study type), participants (demographics) and studied intervention, if applicable. As this study aimed to map evidence and gaps, no attempt to assess risk of bias, assess quality or undertake critical appraisal was made.

### Data synthesis

The reviewers drew upon several established frameworks within EMS and health inequalities literature to structure the map in line with recommendations for evidence and gap maps (Snilstveit et al., 2016). Studies that described health inequalities within EMS were mapped to the framework of EMS care interventions and CORE20PLUS5 outcomes. FB developed the list of EMS care interventions from the WHO emergency care system framework (World Health Organization, 2018): activation of EMS (call and triage); response performance (dispatch); and assessment, treatment and transport to ED or other facilities (telephone or face-to-face care). The visual map was constructed using the framework categories, with bubbles representing the number of studies within each category. Studies that described a specific intervention to reduce health inequalities were mapped separately against the activities in the UK Allied Health Professionals Public Health Strategic Framework (Hindle & Charlesworth, 2019).

## Results

**Figure 1:**
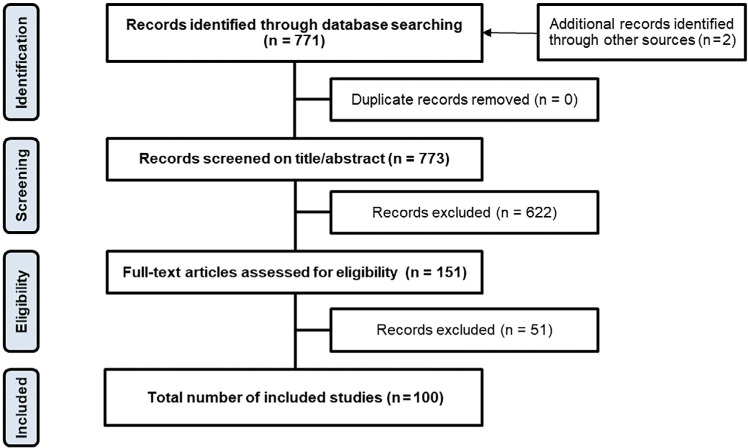
Figure 1. PRISMA flow diagram.

Our database search identified 771 records; the authors included two additional records. Of this total, 671 were excluded due to not fulfilling the inclusion criteria, and 100 studies were included in the evidence map (Figure 1). The most frequent types of study included were retrospective cross-sectional or cohort studies (n = 77). Only nine studies used prospectively collected data, either from a trial intervention or a secondary analysis of trial data, with eight studies identified as modelling studies. Four qualitative studies were included, and one study was described as mixed methods.

The included studies were from the US (n = 63), UK (n = 13), Australia (n = 7), Canada (n = 7), New Zealand (n = 7), Ireland (n = 2) and Portugal (n = 1). The main clinical topic was out-of-hospital cardiac arrest (OHCA) (n = 25), with others focusing on trauma (n = 16), cardiac conditions (n = 14) and stroke (n = 11). Fewer studies involved overdose (n = 3), acute pain (n = 3), falls (n = 1) and pregnancy (n = 1). Some had no particular clinical focus. Most studies focused on adults but n = 9 involved paediatrics.

Similar to the clinical topics represented in the studies overall, UK-based studies predominantly focused on OHCA (n = 3) and trauma (n = 4), with a smaller number of studies exploring cardiac conditions (n = 1) and acute pain (n = 2). In addition, two papers related to mental health and diabetes, which were not represented in the non-UK literature.

**Figure 2:**
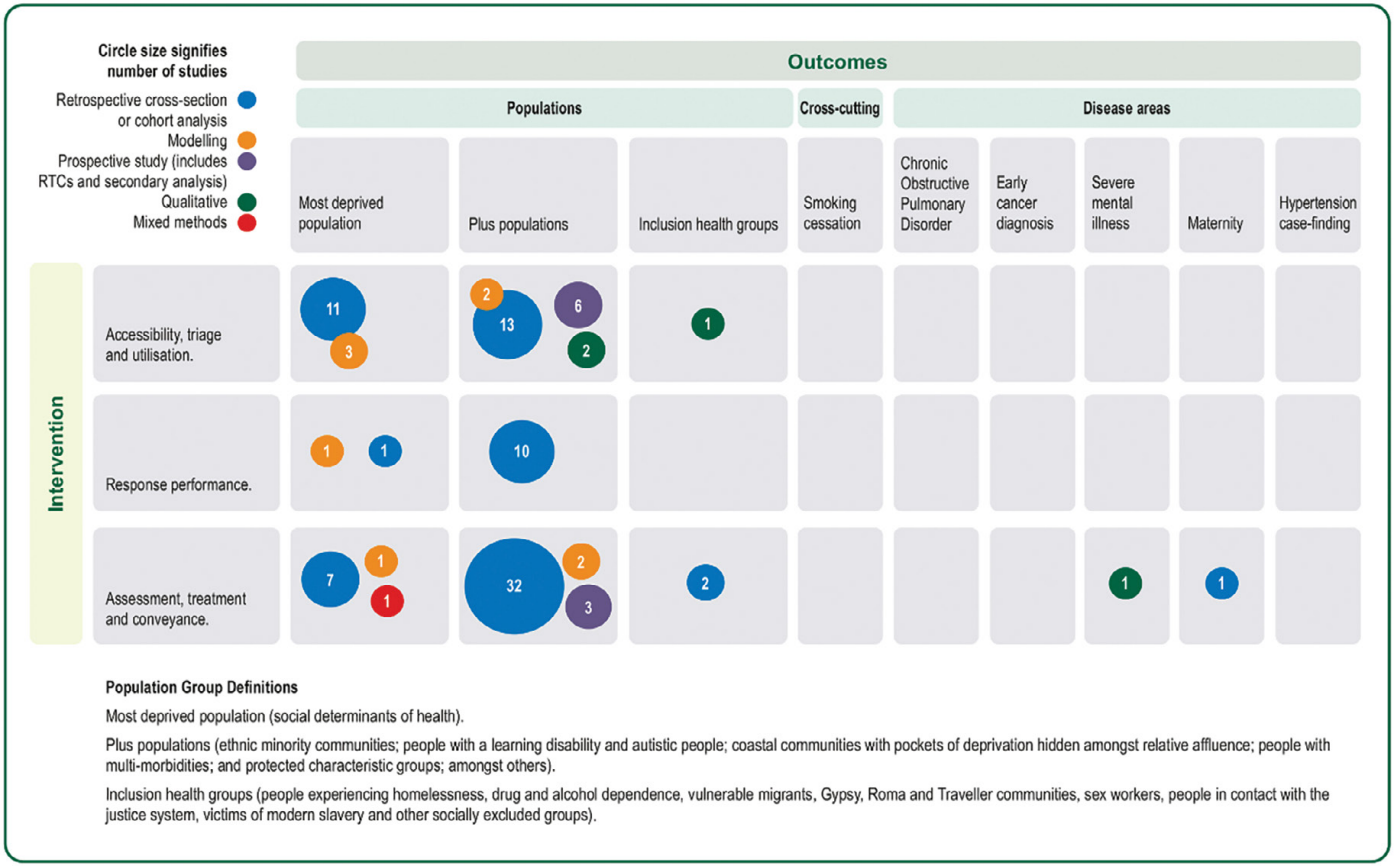
Figure 2. Evidence and gap map framework describing the availability of evidence for EMS care interventions and Core20PLUS5 outcomes, with the circle size signifying the number of studies by study type.

The evidence and gap map in Figure 2 visually demonstrates that most of the evidence is located within PLUS populations, with deprived populations and inclusion health groups receiving much less attention from a research perspective. Interestingly, UK studies focused more on the most deprived 20% of the national population (n = 10) and less on specific PLUS populations, e.g. minoritised ethnic groups (n = 2) and older adults (n = 1), with no UK studies looking at inclusion health groups.

### EMS accessibility, triage and utilisation

The accessibility, triage and utilisation intervention group included a broad range of studies (n = 38). Vogel et al. (2022) identified higher rates of EMS calls in the US in deprived areas and those with high ethnic minority populations. Using the example of stroke in the US, a large registry study found that time from onset of the first stroke symptom to an emergency call was independent of racial, ethnic or sex differences (Gardener et al., 2019), whereas a survey of suspected stroke patients indicated higher rates of EMS calls in African Americans (Malek et al., 2014), with a different survey specifically of African Americans indicating several barriers in the activation of EMS for stroke symptoms, despite higher rates of calls (Skolarus et al., 2011).

Studies related to the links in the OHCA chain of survival (Nolan et al., 2006) predominated in this group (n = 11), including bystander cardiopulmonary resuscitation (CPR) and community defibrillator access and application. Studies from the US showed that emergency call-takers took longer to recognise OHCA in non-English speakers (Bradley et al., 2011) and that people in predominantly Hispanic neighbourhoods (Blewer et al., 2020) and women (Souers et al., 2021) were less likely to receive bystander CPR. In the UK, patients in more deprived areas were less likely to receive bystander CPR (Barnard et al., 2019) or have access to automated external defibrillators (Brown et al., 2022; Leung et al., 2021). This reduced availability of automated external defibrillators was reflected in rural areas in the US (Jadhav & Gaddam, 2021), with the odds of surviving an OHCA being lower in rural areas (Gul et al., 2020; Hanchate et al., 2019; Jarman et al., 2019). Another US study found that female patients experience a lower chance of bystander defibrillator usage, potentially due to bystander fears of it being viewed as sexual assault and public embarrassment (Jadhav & Gaddam, 2021).

This category also contained studies that explored the likelihood of patients calling or requiring an ambulance. The risk of different types of traumatic injury in one area of Australia varied by urban and rural location, with increasing rates of injury in the most disadvantaged areas (Beck et al., 2022). Across the UK, Canada and Australia, living in more deprived areas was linked with higher reported incidences of chest pain (Dawson et al., 2022), children with traumatic injuries (Wohlgemut et al., 2013), stabbings in males (Vulliamy et al., 2018) and violent injury (Cusimano et al., 2010). Deprivation, along with other factors such as ethnicity, sex and age, was linked to OHCA incidence in a Canadian study (Allan et al., 2020), as well as to overall EMS call rates in the US (Vogel et al., 2022). In one Canadian study, areas without ambulance provision were shown to be over-represented by indigenous people indicating an accessibility imbalance (Clark et al., 2012). Modelling of geospatial access showed lower provision of EMS in areas with low population density in New Zealand (Lilley et al., 2019) and socio-economic deprivation in Portugal (Silva & Padeiro, 2020). Arrival at the emergency department by transport methods other than emergency ambulance for patients with chest pain in the US and New Zealand was more likely for younger patients, Latino or other ethnic minorities and those living with a significant other (Garofalo et al., 2012; Neil et al., 2015; Zègre-Hemsey et al., 2016).

### Response performance

Studies describing ambulance response performance represented dispatch disposition and time to ambulance arrival (n = 12). The only UK study looking at response performance concluded that older people with traumatic injuries were less likely to be triaged for dispatch of helicopter EMS, despite requiring interventions provided by helicopter EMS (Griggs et al., 2021). Similarly, lower access to trauma care within an hour was reported for areas with lower income and rurality in a geospatial modelling study from the US (Carr et al., 2017). Longer response times across clinical conditions for areas of high deprivation and rurality were also described in other studies from the US, New Zealand and Ireland (Ashburn et al., 2022; Byrne et al., 2019; Cui et al., 2020, 2021; Garofalo et al., 2012; He et al., 2019; Masterson et al., 2015).

Alongside poorer EMS response performance for low-income areas, Hsia et al. (2018) noted that in the US these areas had fewer white patients, suggesting patients from black and ethnic minority groups were experiencing longer waits for ambulances. However, in another US study, Rao et al. (2021) suggested that rather than a racial bias in EMS performance, variation was due to spatial distributions by race. Similarly, Zachrison et al. (2021) in their US study found no difference in ambulance time performance for patients with non-English language preferences. In a further US study, David and Harrington (2010) reported little or no evidence of race-related disparities in EMS response time; however, the authors noted that African Americans with cardiac problems were more likely to die before EMS arrival.

### Ambulance assessment and treatment

Once attended by an ambulance, longer on-scene times were reported for older people and for females in the US (Aguilar et al., 2012; Ordoobadi et al., 2022). There also appeared to be disparities in analgesia administration based on age and ethnicity in the US and Australia (Kennel et al., 2019; Lord et al., 2016), with a UK study suggesting high deprivation as a factor predicting poor pain management in children (Whitley et al., 2020). Female patients were also found to be less likely to receive the required EMS care package when presenting with acute myocardial infarction compared to their male counterparts in several US and Canadian studies (Bush et al., 2013; Johnson et al., 2014; Safdar et al., 2014; Tataris et al., 2015).

One study from the US indicated that pre-hospital provider recognition of stroke was lower in females and non-white populations when compared to the gold standard of the emergency department diagnosis (Govindarajan et al., 2015); although Zachrison et al. (2021) reported no difference in stroke symptom recognition for patients with non-English language preference in their US study.

In the scope of OHCA care, EMS providers in the US were found to be less likely to use defibrillation or give CPR to patients of black ethnicity (Wilde et al., 2012), whereas in the UK, patients were less likely to receive bystander CPR in more deprived areas (Barnard et al., 2019). With regard to transportation in OHCA without return of spontaneous circulation, males in the US were more likely to be transported to a hospital than females (David & Harrington, 2010).

In relation to non-conveyance decisions, patients with high levels of deprivation were more likely to be conveyed to a hospital following ambulance attendance for lower acuity presentation in the US (Riney et al., 2019) and UK (O’Cathain et al., 2018; van Woerden et al., 2021). A sample of diabetic emergencies in the UK suggested that patients of male sex and facing high levels of deprivation experienced a higher rate of (potentially avoidable) conveyance (van Woerden et al., 2021). Several studies from the US, Canada and New Zealand found a lower likelihood of transport to a specialist receiving facility with increased age, ethnic minority and female sex, such as, for example, trauma centres (Escobar et al., 2022; Gomez et al., 2012; Ryb & Dischinger, 2011; Scheetz & Orazem, 2020) or primary percutaneous coronary intervention services (Dicker et al., 2019; Shen & Hsia, 2016). Hanchate et al. (2019) supported this by identifying that patients were transported to different US hospitals depending on their racial groups, with a UK study similarly indicating that patients from ethnic minorities presenting with pain were less likely to be conveyed to hospital (Asghar et al., 2016).

### Activities that reduce health inequalities

Our searches retrieved five studies that reported on activities aiming to reduce health inequalities within ambulance service settings. Studies were from the US (n = 3), Canada (n = 1) and New Zealand (n = 1), with the data collection period varying in duration from one to seven years (median three years). No studies were identified from the UK. Study types were randomised controlled trials (n = 2), a pilot study (n = 1) and cohort studies (n = 2), with the clinical topic being stroke (n = 3), OHCA (n = 1) or no particular clinical topic (n = 1). All studies focused on ‘PLUS populations’, such as limited English proficiency (n = 2), ethnicity (n = 2) and age (n = 1). However, the study focusing on age also addressed smoking status, hypertension and cancer risk, key clinical areas within the CORE20PLUS5 framework.

Studies incorporated population healthcare (n = 3), health protection (n = 1) and health improvement (n = 2) interventions, with Agarwal et al. (2019) being allocated to multiple categories due to being multi-faceted. Population healthcare interventions involved implementing a new telephone CPR strategy (Sanko et al., 2021), using a dedicated Spanish language line for enrolment in a research trial (Sanossian et al., 2017) and a community-engaged approach to stroke preparedness targeted to under-served black communities (Boden-Albala et al., 2014). Results from these studies were an improvement in the performance of telephone CPR (Sanko et al., 2021), greater enrolment of Hispanics in a research trial (Sanossian et al., 2017) and an increase in stroke cases arriving at the hospital within 4.5 hours of symptom onset (Boden-Albala et al., 2014). The Agarwal et al. (2019) study in the health protection category involved community paramedics leading risk assessments, disease prevention and health promotion sessions, which resulted in improved participant blood pressure readings. Other facets from the Agarwal et al. (2019) study were classed as health improvement because community paramedics led sessions counselling attendees on specific lifestyle changes and relevant, accessible community resources, which resulted in a decrease in EMS calls, alongside gains to quality-adjusted life years and health-related quality of life for the participants. The remaining study within the health improvement category was a stroke campaign with ‘boosters’ for the Maori and Pasifika population, which improved the identification of speech and arm weakness as a stroke sign from 71.7% (774 of 1079 participants) to 75.9% (943 of 1242 participants) (Gordon et al., 2019).

## Discussion

Our evidence and gap map suggests that most of the current published evidence on EMS health inequalities is located within PLUS populations, with deprived populations and inclusion health groups receiving much less attention from a research perspective. The prevalence and presentation of some health conditions may be different for deprived populations and inclusion health groups presenting to EMS, who may be using the service as a last resort to access healthcare (Noulas et al., 2018; Peacock & Peacock, 2006; Peconi et al., 2017). A concern that the five key clinical areas from the Core20PLUS5 framework may not be entirely applicable to the ambulance service setting appear to be corroborated, as they are not represented particularly well in the published literature here.

Although our synthesis is broken down into categories representing EMS access, response and treatment, it should be recognised that these categories are not standalone but are part of the whole patient journey. Patients at risk of health inequalities are probably experiencing poorer EMS care across all these categories in a cumulative fashion. Similarly, characteristics that put patients at risk of health inequalities within EMS care may be cumulative if we believe studies from other settings that suggest cumulative inequality in early childhood adversity and later-life academic achievement, socio-economic status and job security (Jackson, 2015; Shi & Wu, 2020).

A study of routine ambulance data exploring variation in EMS non-conveyance in English ambulance services identified age, sex and social deprivation as influencing rates of non-conveyance at a statistically significant level (O’Cathain et al., 2018). The authors summarised that patient factors are unmodifiable, which is true. However, we postulate that policy informed by ambulance data and knowledge may change the health inequalities experienced by patients due to age, sex, social deprivation and other characteristics discussed in this review.

Rural/urban was a key area of inequality in our included studies but does not explicitly feature in the Core25PLUS framework. This may be an EMS-specific category that requires further investigation, although we should note that rurality as discussed in many of the US and Australian studies (Clark et al., 2012; He et al., 2019) is probably not directly applicable to the UK context given our different geography and population density. Similarly, there is a need to assess the relevance of some of the other findings to the UK population, where the healthcare system, in contrast to the US, is free at the point of access, and there is no indigenous population. Overall, there were fewer UK studies than those from the US, which could be a limitation of the search terms used or reflective of limited UK research in this area.

The clinical focus of included studies was mainly OHCA, trauma and acute myocardial infarction, which only represent a small volume of the work of emergency ambulance staff in the UK. The recent US-focused scoping review by Farcas et al. (2022) also identified an over-representation of OHCA studies, which is traditionally seen as the core role of EMS. Approaches such as the Canadian paramedicine model may be suitable for building capacity to respond to the urgent care workload, while also addressing identified health inequalities.

### Implications and recommendations

The Health and Care Act 2022 (UK Government, 2022) requires NHS organisations to consider the effects of their decision-making concerning population health and wellbeing, the quality of health services for all individuals and the sustainable use of NHS resources. The NHS Operational Planning Guidance for 2023/24 (NHS England, 2023b) builds on the priorities of the Health and Care Act 2022 by highlighting the importance of reflecting the Core20PLUS5 approaches for adults, children and young people in plans and taking a quality improvement approach to addressing health inequalities. Alongside this, statutory requirements such as NHS England’s Equality Delivery System 2022 Patient Safety Incident Response Framework and Statement on information on health inequalities (duty under section 13SA of the National Health Service Act 2006) place a requirement on all NHS organisations, including ambulance services, to proactively reduce the health inequalities that exist within their populations.

Our evidence and gap map indicates that more UK-based research exploring health inequalities of EMS patients is needed to support ambulance service policy and intervention development to reduce health inequality in urgent and emergency care delivery. An example of this may be to use modelling studies to show where best to place EMS resources to reduce rural or socio-economic deprivation inequity of accessibility. Further examples may include community paramedics or community-engaged targeted approaches for specific conditions such as stroke, OHCA or cardiac chest pain to improve population healthcare and address health protection and improvement overall.

### Strengths and limitations

This study is inherently limited by the quality of the reviewed literature and its evidence and gap map methodology. Our review sought to identify where the published knowledge sits rather than the quality of the evidence; therefore, we did not assess the quality of included studies and cannot draw any conclusions about the quality of the existing literature in this area. As with all reviews of published evidence, our review is subject to publication bias, as indicated by all five interventional studies demonstrating positive effects. Our review involved searching one electronic database only. It addressed a broad topic area (i.e. health inequalities) poorly indexed in electronic databases, so our systematic search may not have identified all relevant articles. We did not record reasons for exclusion during the full-text review phase and were unable to include this in the PRISMA flowchart. One reason for exclusion was manuscripts not written in English, due to a lack of resources for translation, which we acknowledge may have biased our findings. Although exploring a patient issue, the review team did not undertake any patient and public involvement and engagement in the design and conduct of this study, which is a limitation. During dissemination and planning of subsequent research studies building on this work, we have planned to include diverse representation in the study team, not just from ambulance staff and public health experts (as was the case for this review) but also from patients and the public, particularly from those who are at risk or have first-hand experience of health inequalities within ambulance service care.

## Conclusions

In summary, this review demonstrated that more UK-based research to explore health inequalities of EMS patients is needed to support ambulance service policy and intervention development to reduce health inequality in urgent and emergency care delivery. The limited published evidence of activities that aim to reduce health inequalities within ambulance service care is promising and invites further innovative ideas that should include community engagement at the forefront to tackle this critical issue of health inequalities.

## Acknowledgements

We acknowledge the support of the Association of Ambulance Chief Executives in generating Figure 2.

## Author contributions

RC and FB jointly conceived the study. EM and RB screened and reviewed the studies for inclusion. FB and CW performed data extraction and synthesis. FB developed the evidence and gap map and prepared the first draft of the manuscript. All authors contributed substantially to its revisions and approved the final manuscript. FB acts as the guarantor for this article.

## Conflict of interest

CW is an associate editor for the *British Paramedic Journal*. RC is National Lead for Public Health, Association of Ambulance Chief Executives.

## Ethics

No formal ethics approval was required for this study, as it was a review of published literature and did not involve human participants.

## Funding

The study received funding from the Association of Ambulance Chief Executives for open access fees.

## References

[bibr_1] Agarwal G. Angeles R. Pirrie M. McLeod B. Marzanek F. Parascandalo J. & Thabane L. (2019). Reducing 9-1-1 emergency medical service calls by implementing a community paramedicine program for vulnerable older adults in public housing in Canada: A multi-site cluster randomized controlled trial. *Prehospital Emergency Care*, 23(5), 718–729. https://doi.org/10.1080/10903127.2019.1566421. 30624150 10.1080/10903127.2019.1566421

[bibr_2] Aguilar S. A. Patel M. Castillo E. Patel E. Fisher R. Ochs G. . . . Dunford J. V. (2012). Gender differences in scene time, transport time, and total scene to hospital arrival time determined by the use of a prehospital electrocardiogram in patients with complaint of chest pain. *Journal of Emergency Medicine*, 43(2), 291–297. https://doi.org/10.1016/j.jemermed.2011.06.130. 22325551 10.1016/j.jemermed.2011.06.130

[bibr_3] Allan K. S. Ray J. G. Gozdyra P. Morrison L. J. Kiss A. Buick J. E. . . . Dorian P. (2020). High risk neighbourhoods: The effect of neighbourhood level factors on cardiac arrest incidence. *Resuscitation*, 149, 100–108. https://doi.org/10.1016/j.resuscitation.2020.02.002. 32068027 10.1016/j.resuscitation.2020.02.002

[bibr_4] Asghar Z. Phung V. H. & Siriwardena A. N. (2016). Ethnicity and pre-hospital care for people with suspected cardiac pain: Cross-sectional study. *Journal of Evaluation in Clinical Practice*, 22(5), 721–725. https://doi.org/10.1111/jep.12523. 26968133 10.1111/jep.12523 PMC5069611

[bibr_5] Ashburn N. P. Snavely A. C. Angi R. M. Scheidler J. F. Crowe R. P. McGinnis H. D. . . . Stopyra J. P. (2022). Prehospital time for patients with acute cardiac complaints: A rural health disparity. *American Journal of Emergency Medicine*, 52, 64–68. https://doi.org/10.1016/j.ajem.2021.11.038. 34871845 10.1016/j.ajem.2021.11.038 PMC9029257

[bibr_6] Barnard E. B. G. Sandbach D. D. Nicholls T. L. Wilson A. W. & Ercole A. (2019). Prehospital determinants of successful resuscitation after traumatic and non-traumatic out-of-hospital cardiac arrest. *Emergency Medicine Journal*, 36(6), 333–339. https://doi.org/10.1136/emermed-2018-208165. 31003991 10.1136/emermed-2018-208165 PMC6582713

[bibr_7] Beck B. Zammit-Mangion A. Fry R. Smith K. & Gabbe B. (2022). Spatiotemporal mapping of major trauma in Victoria, Australia. *PLoS One*, 17(7), e0266521. https://doi.org/10.1371/journal.pone.0266521. 35793336 10.1371/journal.pone.0266521 PMC9258853

[bibr_8] Blewer A. L. Schmicker R. H. Morrison L. J. Aufderheide T. P. Daya M. Starks M. A. . . . Abella B. S. (2020). Variation in bystander cardiopulmonary resuscitation delivery and subsequent survival from out-of-hospital cardiac arrest based on neighborhood-level ethnic characteristics. *Circulation*, 141(1), 34–41. https://doi.org/10.1161/circulationaha.119.041541. 31887076 10.1161/CIRCULATIONAHA.119.041541 PMC6993941

[bibr_9] Boden-Albala B. Edwards D. F. St Clair S. Wing J. J. Fernandez S. Gibbons M. C. . . . Kidwell C. S. (2014). Methodology for a community-based stroke preparedness intervention: The Acute Stroke Program of Interventions Addressing Racial and Ethnic Disparities Study. *Stroke*, 45(7), 2047–2052. https://doi.org/10.1161/strokeaha.113.003502. 24876243 10.1161/STROKEAHA.113.003502 PMC4133149

[bibr_10] Bradley S. M. Fahrenbruch C. E. Meischke H. Allen J. Bloomingdale M. & Rea T. D. (2011). Bystander CPR in out-of-hospital cardiac arrest: The role of limited English proficiency. *Resuscitation*, 82(6), 680–684. https://doi.org/10.1016/j.resuscitation.2011.02.006. 21388734 10.1016/j.resuscitation.2011.02.006

[bibr_11] Brown T. P. Perkins G. D. Smith C. M. Deakin C. D. & Fothergill R. (2022). Are there disparities in the location of automated external defibrillators in England? *Resuscitation*, 170, 28–35. https://doi.org/10.1016/j.resuscitation.2021.10.037. 34757059 10.1016/j.resuscitation.2021.10.037 PMC8786665

[bibr_12] Bush M. Glickman L. T. Fernandez A. R. Garvey J. L. & Glickman S. W. (2013). Variation in the use of 12-lead electrocardiography for patients with chest pain by emergency medical services in North Carolina. *Journal of the American Heart Association*, 2(4), e000289. https://doi.org/10.1161/jaha.113.000289. 23920232 10.1161/JAHA.113.000289 PMC3828790

[bibr_13] Byrne J. P. Mann N. C. Dai M. Mason S. A. Karanicolas P. Rizoli S. & Nathens A. B. (2019). Association between emergency medical service response time and motor vehicle crash mortality in the United States. *JAMA Surg*, 154(4), 286–293. https://doi.org/10.1001/jamasurg.2018.5097. 30725080 10.1001/jamasurg.2018.5097 PMC6484802

[bibr_14] Carr B. G. Bowman A. J. Wolff C. S. Mullen M. T. Holena D. N. Branas C. C. & Wiebe D. J. (2017). Disparities in access to trauma care in the United States: A population-based analysis. *Injury*, 48(2), 332–338. https://doi.org/10.1016/j.injury.2017.01.008. 28069138 10.1016/j.injury.2017.01.008 PMC5292279

[bibr_15] Carter L. (2018). *Operational productivity and performance in English NHS ambulance trusts: Unwarranted variations*. https://www.england.nhs.uk/wp-content/uploads/2019/09/Operational_productivity_and_performance_NHS_Ambulance_Trusts_final.pdf.

[bibr_16] Clark R. A. Coffee N. Turner D. Eckert K. A. van Gaans D. Wilkinson D. . . . Tonkin A. M. (2012). Application of geographic modeling techniques to quantify spatial access to health services before and after an acute cardiac event: The Cardiac Accessibility and Remoteness Index for Australia (ARIA) project. *Circulation*, 125(16), 2006–2014. https://doi.org/10.1161/circulationaha.111.083394. 22451583 10.1161/CIRCULATIONAHA.111.083394

[bibr_17] Cui E. R. Beja-Glasser A. Fernandez A. R. Grover J. M. Mann N. C. & Patel M. D. (2020). Emergency medical services time intervals for acute chest pain in the United States, 2015–2016. *Prehospital Emergency Care*, 24(4), 557–565. https://doi.org/10.1080/10903127.2019.1676346. 31580176 10.1080/10903127.2019.1676346

[bibr_18] Cui E. R. Fernandez A. R. Zegre-Hemsey J. K. Grover J. M. Honvoh G. Brice J. H. . . . Patel M. D. (2021). Disparities in emergency medical services time intervals for patients with suspected acute coronary syndrome: Findings from the North Carolina Prehospital Medical Information System. *Journal of the American Heart Association*, 10(15), e019305. https://doi.org/10.1161/jaha.120.019305. 34323113 10.1161/JAHA.120.019305 PMC8475668

[bibr_19] Cusimano M. Marshall S. Rinner C. Jiang D. & Chipman M. (2010). Patterns of urban violent injury: A spatio-temporal analysis. *PLoS One*, 5(1), e8669. https://doi.org/10.1371/journal.pone.0008669. 20084271 10.1371/journal.pone.0008669 PMC2800193

[bibr_20] David G. & Harrington S. E. (2010). Population density and racial differences in the performance of emergency medical services. *Journal of Health Economics*, 29(4), 603–615. https://doi.org/10.1016/j.jhealeco.2010.03.004. 20398954 10.1016/j.jhealeco.2010.03.004

[bibr_21] Dawson L. P. Andrew E. Nehme Z. Bloom J. Biswas S. Cox S. . . . Stub D. (2022). Association of socioeconomic status with outcomes and care quality in patients presenting with undifferentiated chest pain in the setting of universal health care coverage. *Journal of the American Heart Association*, 11(7), e024923. https://doi.org/10.1161/jaha.121.024923. 35322681 10.1161/JAHA.121.024923 PMC9075482

[bibr_22] Dicker B. Todd V. F. Tunnage B. Swain A. Smith T. & Howie G. (2019). Direct transport to PCI-capable hospitals after out-of-hospital cardiac arrest in New Zealand: Inequities and outcomes. *Resuscitation*, 142, 111–116. https://doi.org/10.1016/j.resuscitation.2019.06.283. 31271727 10.1016/j.resuscitation.2019.06.283

[bibr_23] Escobar N. DiMaggio C. Frangos S. G. Winchell R. J. Bukur M. Klein M. J. . . . Berry C. (2022). Disparity in transport of critically injured patients to trauma centers: Analysis of the National Emergency Medical Services Information System (NEMSIS). *Journal of the American College of Surgeons*, 235(1), 78–85. https://doi.org/10.1097/xcs.0000000000000230. 35703965 10.1097/XCS.0000000000000230

[bibr_24] Farcas A. M. Joiner A. P. Rudman J. S. Ramesh K. Torres G. Crowe R. P. . . . Haamid A. (2022). Disparities in emergency medical services care delivery in the United States: A scoping review. *Prehospital Emergency Care*, 27(8), 1058–1071. https://doi.org/10.1080/10903127.2022.2142344. 36369725 10.1080/10903127.2022.2142344

[bibr_25] Gardener H. Pepe P. E. Rundek T. Wang K. Dong C. Ciliberti M. . . . Sacco R. L. (2019). Need to prioritize education of the public regarding stroke symptoms and faster activation of the 9-1-1 system: Findings from the Florida-Puerto Rico CReSD Stroke Registry. *Prehospital Emergency Care*, 23(4), 439–446. https://doi.org/10.1080/10903127.2018.1525458. 30239244 10.1080/10903127.2018.1525458 PMC6483889

[bibr_26] Garofalo D. Grey C. Lee M. Exeter D. & Kerr A. J. (2012). Pre-hospital delay in acute coronary syndromes: PREDICT CVD-18. *N Z Med J*, 125(1348), 12–22. 22282273

[bibr_27] Gomez D. Haas B. de Mestral C. Sharma S. Hsiao M. Zagorski B. . . . Nathens A. B. (2012). Gender-associated differences in access to trauma center care: A population-based analysis. *Surgery*, 152(2), 179–185. https://doi.org/10.1016/j.surg.2012.04.006. 22727364 10.1016/j.surg.2012.04.006

[bibr_28] Gordon C. Bell R. & Ranta A. (2019). Impact of the national public ‘FAST’ campaigns. *N Z Med J*, 132(1507), 48–56. 31830016

[bibr_29] Govindarajan P. Friedman B. T. Delgadillo J. Q. Ghilarducci D. Cook L. J. Grimes B. . . . Johnston S. C. (2015). Race and sex disparities in prehospital recognition of acute stroke. *Academic Emergency Medicine*, 22(3), 264–272. https://doi.org/10.1111/acem.12595. 25728356 10.1111/acem.12595 PMC4355063

[bibr_30] Griggs J. E. Barrett J. W. ter Avest E. de Coverly R. Nelson M. Williams J. & Lyon R. M. (2021). Helicopter emergency medical service dispatch in older trauma: Time to reconsider the trigger? *Scandinavian Journal of Trauma, Resuscitation and Emergency Medicine*, 29(1), 62. https://doi.org/10.1186/s13049-021-00877-3. 33962682 10.1186/s13049-021-00877-3 PMC8103626

[bibr_31] Gul S. S. Cohen S. A. Becker T. K. Huesgen K. Jones J. M. & Tyndall J. A. (2020). Patient, neighborhood, and spatial determinants of out-of-hospital cardiac arrest outcomes throughout the chain of survival: A community-oriented multilevel analysis. *Prehospital Emergency Care*, 24(3), 307–318. https://doi.org/10.1080/10903127.2019.1640324. 31287347 10.1080/10903127.2019.1640324 PMC7000295

[bibr_32] Hanchate A. D. Paasche-Orlow M. K. Baker W. E. Lin M. Y. Banerjee S. & Feldman J. (2019). Association of race/ethnicity with emergency department destination of emergency medical services transport. *JAMA Netw Open*, 2(9), e1910816. https://doi.org/10.1001/jamanetworkopen.2019.10816. 31490537 10.1001/jamanetworkopen.2019.10816 PMC6735492

[bibr_33] He Z. Qin X. Renger R. & Souvannasacd E. (2019). Using spatial regression methods to evaluate rural emergency medical services (EMS). *American Journal of Emergency Medicine*, 37(9), 1633–1642. https://doi.org/10.1016/j.ajem.2018.11.029. 30522937 10.1016/j.ajem.2018.11.029

[bibr_34] Hindle L. & Charlesworth L. (2019). *UK AHP public health strategic framework*. http://www.ahpf.org.uk/files/UK%20AHP%20Public%20Health%20Strategic%20Framework%202019-2024.pdf.

[bibr_35] Hsia R. Y. Huang D. Mann N. C. Colwell C. Mercer M. P. Dai M. & Niedzwiecki M. J. (2018). A US national study of the association between income and ambulance response time in cardiac arrest. *JAMA Netw Open*, 1(7), e185202. https://doi.org/10.1001/jamanetworkopen.2018.5202. 30646394 10.1001/jamanetworkopen.2018.5202 PMC6324393

[bibr_36] Jackson M. I. (2015). Cumulative inequality in child health and academic achievement. *Journal of Health and Social Behavior*, 56(2), 262–280. https://doi.org/10.1177/0022146515581857. 25926564 10.1177/0022146515581857 PMC4631384

[bibr_37] Jadhav S. & Gaddam S. (2021). Gender and location disparities in prehospital bystander AED usage. *Resuscitation*, 158, 139–142. https://doi.org/10.1016/j.resuscitation.2020.11.006. 33189804 10.1016/j.resuscitation.2020.11.006

[bibr_38] Jarman M. P. Hashmi Z. Zerhouni Y. Udyavar R. Newgard C. Salim A. & Haider A. H. (2019). Quantifying geographic barriers to trauma care: Urban-rural variation in prehospital mortality. *Journal of Trauma and Acute Care Surgery*, 87(1), 173–180. https://doi.org/10.1097/ta.0000000000002335. 31033885 10.1097/TA.0000000000002335

[bibr_39] Johnson T. J. Schultz B. R. & Guyette F. X. (2014). Characterizing analgesic use during air medical transport of injured children. *Prehospital Emergency Care*, 18(4), 531–538. https://doi.org/10.3109/10903127.2014.916018. 24878300 10.3109/10903127.2014.916018

[bibr_40] Kennel J. Withers E. Parsons N. & Woo H. (2019). Racial/ethnic disparities in pain treatment: Evidence from Oregon emergency medical services agencies. *Medical Care*, 57(12), 924–929. https://doi.org/10.1097/mlr.0000000000001208. 31730566 10.1097/MLR.0000000000001208

[bibr_41] Leung K. H. B. Brooks S. C. Clegg G. R. & Chan T. C. Y. (2021). Socioeconomically equitable public defibrillator placement using mathematical optimization. *Resuscitation*, 166, 14–20. https://doi.org/10.1016/j.resuscitation.2021.07.002. 34271132 10.1016/j.resuscitation.2021.07.002

[bibr_42] Lilley R. de Graaf B. Kool B. Davie G. Reid P. Dicker B. . . . Branas C. (2019). Geographical and population disparities in timely access to prehospital and advanced level emergency care in New Zealand: A cross-sectional study. *BMJ Open*, 9(7), e026026. https://doi.org/10.1136/bmjopen-2018-026026. 10.1136/bmjopen-2018-026026 PMC6661642 31350239

[bibr_43] Lord B. Jennings P. A. & Smith K. (2016). The epidemiology of pain in children treated by paramedics. *Emergency Medicine Australasia*, 28(3), 319–324. https://doi.org/10.1111/1742-6723.12586. 27147481 10.1111/1742-6723.12586

[bibr_44] Malek A. M. Adams R. J. Debenham E. Boan A. D. Kazley A. S. Hyacinth H. I. . . . Lackland D. T. (2014). Patient awareness and perception of stroke symptoms and the use of 911. *Journal of Stroke & Cerebrovascular Diseases*, 23(9), 236212371. https://doi.org/10.1016/j.jstrokecerebrovasdis.2014.05.011. 10.1016/j.jstrokecerebrovasdis.2014.05.011 PMC4180792 25213451

[bibr_45] Masterson S. Wright P. O’Donnell C. Vellinga A. Murphy A. W. Hennelly D. . . . Deasy C. (2015). Urban and rural differences in out-of-hospital cardiac arrest in Ireland. *Resuscitation*, 91, 42–47. https://doi.org/10.1016/j.resuscitation.2015.03.012. 25818707 10.1016/j.resuscitation.2015.03.012

[bibr_46] Miake-Lye I. M. Hempel S. Shanman R. & Shekelle P. G. (2016). What is an evidence map? A systematic review of published evidence maps and their definitions, methods, and products. *Systematic Reviews*, 5, 28. https://doi.org/10.1186/s13643-016-0204-x. 26864942 10.1186/s13643-016-0204-x PMC4750281

[bibr_47] Neil W. P. Raman R. Hemmen T. M. Ernstrom K. Meyer B. C. Meyer D. M. & Ovbiagele B. (2015). Association of Hispanic ethnicity with acute ischemic stroke care processes and outcomes. *Ethnicity and Disease*, 25(1), 19–23. 25812247

[bibr_48] NHS Confederation. (2022). *The unequal impact of COVID-19: Investigating the effect on people with certain protected characteristics*. https://www.nhsconfed.org/publications/unequal-impact-covid-19-protected-characteristics.

[bibr_49] NHS Digital. (2023). *Ambulance clinical outcomes (AmbCO)*. https://digital.nhs.uk/data-and-information/data-collections-and-data-sets/data-collections/ambco.

[bibr_50] NHS England. (2021). *Core20PLUS5 (adults) – an approach to reducing healthcare inequalities*. https://www.england.nhs.uk/about/equality/equality-hub/national-healthcare-inequalities-improvement-programme/core20plus5/.

[bibr_51] NHS England. (2023a). *What are healthcare inequalities*? https://www.england.nhs.uk/about/equality/equality-hub/national-healthcare-inequalities-improvement-programme/what-are-healthcare-inequalities/.

[bibr_52] NHS England. (2023b). *2023/24 priorities and operational planning guidance*. https://www.england.nhs.uk/wp-content/uploads/2022/12/PRN00021-23-24-priorities-and-operational-planning-guidance-v1.1.pdf.

[bibr_53] Nolan J. Soar J. & Eikeland H. (2006) The chain of survival. *Resuscitation*, 71(3), 270–271. https://pubmed.ncbi.nlm.nih.gov/17070646/. 17070646 10.1016/j.resuscitation.2006.09.001

[bibr_54] Noulas A. Moffatt C. Hristova D. & Gonçalves B. (2018). *Foursquare to the rescue: Predicting ambulance calls across geographies*. DH ‘18: Proceedings of the 2018 International Conference on Digital Health, Lyon, France.

[bibr_55] O’Cathain A. Knowles E. Bishop-Edwards L. Coster J. Crum A. Jacques R. . . . Williams J. (2018). Understanding variation in ambulance service non-conveyance rates: A mixed methods study. *NIHR Health Services and Delivery Research*. https://doi.org/10.3310/hsdr06190. 29870196

[bibr_56] Ordoobadi A. J. Peters G. A. Westfal M. L. Kelleher C. M. & Chang D. C. (2022). Disparity in prehospital scene time for geriatric trauma patients. *American Journal of Surgery*, 223(6), 1200–1205. https://doi.org/10.1016/j.amjsurg.2021.10.031. 34756693 10.1016/j.amjsurg.2021.10.031

[bibr_57] Peacock P. J. & Peacock J. L. (2006). Emergency call work-load, deprivation and population density: An investigation into ambulance services across England. *Journal of Public Health (Oxford)*, 28(2), 111–115. https://doi.org/10.1093/pubmed/fdi079. 10.1093/pubmed/fdi079 16531473

[bibr_58] Peconi J. Macey S. Rodgers S. Russell I. Snooks H. & Watkins A. (2017). Advice given by NHS Direct in Wales: Do deprived patients get more urgent decisions? Study of routine data. *Journal of Epidemiology and Community Health*, 71(9), 849–856. https://doi.org/10.1136/jech-2017-208978. 28733459 10.1136/jech-2017-208978 PMC5561357

[bibr_59] Rao N. Chang J. & Paydarfar D. (2021). Characterizing the performance of emergency medical transport time metrics in a residentially segregated community. *American Journal of Emergency Medicine*, 50, 111–119. https://doi.org/10.1016/j.ajem.2021.07.013. 34340164 10.1016/j.ajem.2021.07.013

[bibr_60] Riney L. C. Brokamp C. Beck A. F. Pomerantz W. J. Schwartz H. P. & Florin T. A. (2019). Emergency medical services utilization is associated with community deprivation in children. *Prehospital Emergency Care*, 23(2), 225–232. https://doi.org/10.1080/10903127.2018.1501124. 30118621 10.1080/10903127.2018.1501124

[bibr_61] Ryb G. E. & Dischinger P. C. (2011). Disparities in trauma center access of older injured motor vehicular crash occupants. *Journal of Trauma*, 71(3), 742–747. https://doi.org/10.1097/TA.0b013e31822ba010. 21909004 10.1097/TA.0b013e31822ba010

[bibr_62] Safdar B. Stolz U. Stiell I. G. Cone D. C. Bobrow B. J. deBoehr M. . . . Spaite D. W. (2014). Differential survival for men and women from out-of-hospital cardiac arrest varies by age: Results from the OPALS study. *Academic Emergency Medicine*, 21(12), 1503–1511. https://doi.org/10.1111/acem.12540. 25491713 10.1111/acem.12540

[bibr_63] Sanko S. Feng S. Lane C. & Eckstein M. (2021). Comparison of emergency medical dispatch systems for performance of telecommunicator-assisted cardiopulmonary resuscitation among 9-1-1 callers with limited English proficiency. *JAMA Netw Open*, 4(6), e216827. https://doi.org/10.1001/jamanetworkopen.2021.6827. 34076700 10.1001/jamanetworkopen.2021.6827 PMC8173370

[bibr_64] Sanossian N. Rosenberg L. Liebeskind D. S. Starkman S. Eckstein M. Stratton S. . . . Saver J. L. (2017). A dedicated Spanish language line increases enrollment of Hispanics into prehospital clinical research. *Stroke*, 48(5), 1389–1391. https://doi.org/10.1161/strokeaha.117.014745. 28389617 10.1161/STROKEAHA.117.014745

[bibr_65] Saran A. & White H. (2018). Evidence and gap maps: A comparison of different approaches. *Campbell Syst Rev*, 14(1), 1–38. https://doi.org/10.4073/cmdp.2018.2. 10.4073/cmdp.2018.2 PMC8428058 37131398

[bibr_66] Scheetz L. J. & Orazem J. P. (2020). The influence of sociodemographic factors on trauma center transport for severely injured older adults. *Health Services Research*, 55(3), 411–418. https://doi.org/10.1111/1475-6773.13270. 31994218 10.1111/1475-6773.13270 PMC7240777

[bibr_67] Shen Y. C. & Hsia R. Y. (2016). Do patients hospitalised in high-minority hospitals experience more diversion and poorer outcomes? A retrospective multivariate analysis of Medicare patients in California. *BMJ Open*, 6(3), e010263. https://doi.org/10.1136/bmjopen-2015-010263. 10.1136/bmjopen-2015-010263 PMC4800138 26988352

[bibr_68] Shi Z. & Wu C. (2020). Early life adversity and health inequality: A dual interaction model. *The Journal of Chinese Sociology*, 7(1), 11. https://doi.org/10.1186/s40711-020-00121-y.

[bibr_69] Silva K. & Padeiro M. (2020). Assessing inequalities in geographical access to emergency medical services in metropolitan Lisbon: A cross-sectional and ecological study. *BMJ Open*, 10, e033777. https://doi.org/10.1136/bmjopen-2019-033777. 10.1136/bmjopen-2019-033777 PMC7651750 33158817

[bibr_70] Skolarus L. E. Zimmerman M. A. Murphy J. Brown D. L. Kerber K. A. Bailey S. . . . Morgenstern L. B. (2011). Community-based participatory research: A new approach to engaging community members to rapidly call 911 for stroke. *Stroke*, 42(7), 1862–1866. https://doi.org/10.1161/strokeaha.110.609495. 21617148 10.1161/STROKEAHA.110.609495 PMC4005877

[bibr_71] Snilstveit B. Vojtkova M. Bhavsar A. Stevenson J. & Gaarder M. (2016). Evidence and gap maps: A tool for promoting evidence informed policy and strategic research agendas. *Journal of Clinical Epidemiology*, 79, 1201–129. https://doi.org/10.1016/j.jclinepi.2016.05.015. 10.1016/j.jclinepi.2016.05.015 27387966

[bibr_72] Souers A. Zuver C. Rodriguez A. Van Dillen C. Hunter C. & Papa L. (2021). Bystander CPR occurrences in out of hospital cardiac arrest between sexes. *Resuscitation*, 166, 1–6. https://doi.org/10.1016/j.resuscitation.2021.06.021. 34237358 10.1016/j.resuscitation.2021.06.021

[bibr_73] Suleman M. Sonthalia S. Webb C. Tinson A. Kane M. Bunbury S. . . . Bibby J. (2021). *Unequal pandemic, fairer recovery: The COVID-19 impact inquiry report*. https://www.health.org.uk/publications/reports/unequal-pandemic-fairer-recovery.

[bibr_74] Tataris K. L. Mercer M. P. & Govindarajan P. (2015). Prehospital aspirin administration for acute coronary syndrome (ACS) in the USA: An EMS quality assessment using the NEMSIS 2011 database. *Emergency Medicine Journal*, 32(11), 876–881. https://doi.org/10.1136/emermed-2014-204299. 25678574 10.1136/emermed-2014-204299

[bibr_75] UK Government. (2022). *Health and Care Act 2022*. https://www.legislation.gov.uk/ukpga/2022/31/contents/enacted.

[bibr_76] van Woerden H. Bucholc M. Clubbs Coldron B. Coates V. Heaton J. McCann M. . . . MacRury S. (2021). Factors influencing hospital conveyance following ambulance attendance for people with diabetes: A retrospective observational study. *Diabetic Medicine*, 38(4), e14384. https://doi.org/https://doi.org/10.1111/dme.14384. 33464629 10.1111/dme.14384

[bibr_77] Vogel J. A. Burnham R. I. McVaney K. Havranek E. P. Edwards D. Hulac S. & Sasson C. (2022). The importance of neighborhood in 9-1-1 ambulance contacts: A geospatial analysis of medical and trauma emergencies in Denver. *Prehospital Emergency Care*, 26(2), 233–245. https://doi.org/10.1080/10903127.2020.1868634. 33400608 10.1080/10903127.2020.1868634

[bibr_78] Vulliamy P. Faulkner M. Kirkwood G. West A. O’Neill B. Griffiths M. P. . . . Brohi K. (2018). Temporal and geographic patterns of stab injuries in young people: A retrospective cohort study from a UK major trauma centre. *BMJ Open*, 8(10), e023114. https://doi.org/10.1136/bmjopen-2018-023114. 10.1136/bmjopen-2018-023114 PMC6231558 30401726

[bibr_79] Welch V. Petticrew M. Tugwell P. Moher D. O’Neill J. Waters E. & White H. (2012). PRISMA-equity 2012 extension: Reporting guidelines for systematic reviews with a focus on health equity. *PLoS Med*, 9(10), e1001333. https://doi.org/10.1371/journal.pmed.1001333. 23222917 10.1371/journal.pmed.1001333 PMC3484052

[bibr_80] Whitley G. A. Hemingway P. Law G. R. Wilson C. & Siriwardena A. N. (2020). Predictors of effective management of acute pain in children within a UK ambulance service: A cross-sectional study. *American Journal of Emergency Medicine*, 38(7), 1424–1430. https://doi.org/10.1016/j.ajem.2019.11.043. 31864872 10.1016/j.ajem.2019.11.043

[bibr_81] Wilde E. T. Robbins L. S. & Pressley J. C. (2012). Racial differences in out-of-hospital cardiac arrest survival and treatment. *Emergency Medicine Journal*, 29(5), 415–419. https://doi.org/10.1136/emj.2010.109736. 21546508 10.1136/emj.2010.109736

[bibr_82] Wohlgemut J. M. Morrison J. J. Apodaca A. N. Egan G. Sponseller P. D. Driver C. P. & Jansen J. O. (2013). Demographic and geographical characteristics of pediatric trauma in Scotland. *Journal of Pediatric Surgery*, 48(7), 1593–1597. https://doi.org/10.1016/j.jpedsurg.2013.03.060. 23895978 10.1016/j.jpedsurg.2013.03.060

[bibr_83] World Health Organization. (2018). *WHO emergency care system framework*. https://www.who.int/publications/i/item/who-emergency-care-system-framework.

[bibr_84] Zachrison K. S. Natsui S. Luan Erfe B. M. Mejia N. I. & Schwamm L. H. (2021). Language preference does not influence stroke patients’ symptom recognition or emergency care time metrics. *American Journal of Emergency Medicine*, 40, 177–180. https://doi.org/10.1016/j.ajem.2020.10.064. 33168382 10.1016/j.ajem.2020.10.064

[bibr_85] Zègre-Hemsey J. K. Pickham D. & Pelter M. M. (2016). Electrocardiographic indicators of acute coronary syndrome are more common in patients with ambulance transport compared to those who self-transport to the emergency department journal of electrocardiology. *Journal of Electrocardiology*, 49(6), 944–950. https://doi.org/10.1016/j.jelectrocard.2016.08.008. 27614946 10.1016/j.jelectrocard.2016.08.008 PMC5159244

